# Predictive Value of Combined Preoperative Carcinoembryonic Antigen Level and Ki-67 Index in Patients With Gastric Neuroendocrine Carcinoma After Radical Surgery

**DOI:** 10.3389/fonc.2021.533039

**Published:** 2021-03-02

**Authors:** Jianwei Xie, YaJun Zhao, Yanbing Zhou, Qingliang He, Hankun Hao, Xiantu Qiu, Gang Zhao, Yanchang Xu, Fangqin Xue, Jinping Chen, Guoqiang Su, Ping Li, Chao-Hui Zheng, Chang-Ming Huang

**Affiliations:** ^1^ Department of Gastric Surgery, Fujian Medical University Union Hospital, Fuzhou, China; ^2^ West District of The First Affiliated Hospital of USTC, Division of Life Sciences and Medicine, University of Science and Technology of China, Anhui, China; ^3^ Department of Gastrointestinal Surgery, Affiliated Hospital of Qingdao University, Qingdao, China; ^4^ Department of Gastrointestinal Surgery, The First Affiliated Hospital of Fujian Medical University, Fuzhou, China; ^5^ Huashan Hospital, Fudan University, Shanghai, China; ^6^ Department of Gastrointestinal Surgery, The Affiliated Hospital of Putian University, Putian, China; ^7^ Renji Hospital, Shanghai Jiaotong University, Shanghai, China; ^8^ Fujian Medicine University Teaching Hospital, The First Hospital of Putian, Putian, China; ^9^ Provincial Clinical Medical College of Fujian Medical University, Fujian Provincial Hospital, Fujian, China; ^10^ Department of Gastrointestinal Surgery, Quanzhou First Hospital Affiliated to Fujian Medical University, Quanzhou, China; ^11^ Department of Gastrointestinal Surgery, Xiamen Cancer Center, First Affiliated Hospital of Xiamen University, Xiamen, China

**Keywords:** gastric neuroendocrine carcinoma, carcinoembryonic antigen (CEA), Ki-67, prognosis, nomogram

## Abstract

**Précis:**

We present a valid and reproducible nomogram that combined the TNM stage as well as the Ki-67 index and carcinoembryonic antigen levels; the nomogram may be an indispensable tool to help predict individualized risks of death and help clinicians manage patients with gastric neuroendocrine carcinoma.

**Background:**

To analyze the long-term outcomes of patients with grade 3 GNEC who underwent curative surgery and investigated whether the combination of carcinoembryonic antigen (CEA) levels and Ki-67 index can predict the prognosis of patients with gastric neuroendocrine carcinoma (GNEC) and constructed a nomogram to predict patient survival.

**Methods:**

In the training cohort, data were collected from 405 patients with GNEC after radical surgery at seven Chinese centers. A nomogram was constructed to predict long-term prognosis. Data for the validation cohort were collected from 305 patients.

**Results:**

The 5-year overall survival (OS) was worse in the high CEA group than in the normal CEA group (40.5% vs. 55.2%, p = 0.013). The 5-year OS was significantly worse in the high Ki-67 index group than in the low Ki-67 index group (47.9% vs. 57.2%, p = 0.012). Accordingly, we divided the whole cohort into a KC(-) group (low Ki-67 index and normal CEA) and KC(+) group (high Ki-67 index and/or high CEA). The KC(+) group had a worse prognosis than the KC(-) group (64.6% vs. 46.8%, p < 0.001). KC(+) and the AJCC 8^th^ stage were independent factors for OS. Then, we combined KC status and the AJCC 8^th^ stage to establish a nomogram; the C-index and area under the curve (AUC) were higher for the nomogram than for the AJCC 8^th^ stage (C-index: 0.660 vs. 0.635, p = 0.005; AUC: 0.700 vs. 0.675, p = 0.020). The calibration curve verified that the nomogram had a good predictive value, with similar findings in the validation groups.

**Conclusions:**

The nomogram based on KC status and the AJCC 8^th^ stage predicted the prognosis of patients with GNEC well.

## Introduction

Gastric neuroendocrine carcinomas (GNECs) are rare and account for 0.2% to 1.5% of all gastric cancers, with increasing incidence in recent years (1–3). GNEC has a highly aggressive malignant propensity for early spread to the lymph nodes and distant organs, reducing the likelihood of curative surgery. Compared with gastric adenocarcinomas, GNECs have a worse survival, with 5-year overall survival (OS) rates ranging from 31% to 38%, even after radical gastrectomy and adjuvant chemotherapy (3, 4).

Currently, the prognosis of GNECs is estimated based on the American Joint Committee on Cancer staging system, which does not factor in prognostic determinants other than the TNM stage (5). However, survival is not uniform because of the differing genetic, cellular, and behavioral characteristics of GNECs. Therefore, by integrating additional significant prognostic factors should be integrated to provide a better assessment of an individual patient’s postoperative survival. Ki-67 index is an important factor to evaluate the grade of GNECs, but its prognostic value in GNECs is still controversial (6, 7). And Previous studies have shown that preoperative tumor markers are important prognostic fators for gastric adenocarcinoma (8), but their prognostic value in GNECs has not been confirmed. In addition, to the best of our knowledge, no clinical tools are available for predicting the OS rate of patients with GNEC.

This study aimed to analyze the long-term outcomes of patients with grade 3 GNEC who underwent curative surgery in eleven large-volume centers in China. The OS and significant predictors for OS were analyzed. Using these predictors, a nomogram that predicted OS was developed to calculate the risk of death of individual patients and to identify high-risk patients after curative resection.

## Methods

### Study Population

In the training cohort, we retrospectively analyzed the data regarding the epidemiological factors, plasma tumor markers, tumor proliferation index, treatment regimens, and survival outcomes of patients diagnosed with GNEC at eleven Chinese university hospitals between October 2006 and August 2018. The inclusion criteria were as follows: (1) those without distant metastasis, as assessed on preoperative examinations; (2) those who underwent D2 lymph node dissection and R0 resection considering the postoperative pathological diagnosis. The exclusion criteria were as follows: (1) those with a preoperatively and intraoperatively confirmed diagnosis of distant metastasis; (2) those who underwent preoperative adjuvant chemotherapy or radiotherapy; (3) those with incomplete clinical data; and (4) those without information about survival data (**Figure S1**). The external validation datasets that satisfied the aforementioned inclusion and exclusion criteria were obtained. The training group included 7 centers from Fujian Province, including the Fujian Medical University Union Hospital (FMUUH), Fujian Provincial Hospital, the First Affiliated Hospital of Fujian Medical University, Putian City First Hospital, Putian College Hospital, Quanzhou City First Hospital, and the First Affiliated Hospital of Xiamen University. The validation group was obtained from 4 centers outside Fujian Province, including Anhui Provincial Hospital, Huashan Hospital affiliated to Fudan University, Affiliated Hospital of Qingdao University, and Shanghai Renji Hospital. All the above-mentioned 11 centers are independent centers. The study was conducted in accordance with the Declaration of Helsinki and the Ethical Guidelines for Clinical Studies, and was approved by the institutional review boards of FMUUH (2018YF031-02).

The tumors were graded based on the Ki-67 index and mitotic index according to the World Health Organization (WHO) guidelines (9). GNEC (2010 WHO grade 3) was defined as a neuroendocrine carcinoma (Ki-67 >20% or mitotic index >20/high-power field [HPF]) or mixed adenoneuroendocrine carcinoma (Ki-67 >20% or mitotic index >20/HPF) (6, 10). The Ki-67 index was obtained from postoperative pathological specimens. Immunohistochemistry for Ki-67 was performed on a BenchMark^®^ XT automated staining system (Ventana Medical Systems, Inc. Tucson, Arizona, USA), by using the horseradish peroxidase (HRP) complex method with a rabbit monoclonal antibody against Ki-67 (Maxvision^TM2^, Fuzhou, Fujian, China) (**Figure S2**).

The preoperative carcinoembryonic antigen (CEA) level was measured within 1 month before surgery by using enzyme immunoassays. The cut-off value for CEA was 5 ng/mL and that for Ki-67 was 60% (11). OS was defined as the time from surgery to death due to any cause or to the time of censoring on the date of the last follow-up. The tumors were staged using TNM staging according to the eighth edition of the Cancer Staging Manual of the American Joint Committee on Cancer (AJCC)/International Union Against Cancer (5, 12).

### Surgical Treatment and Pathological Examination

All surgical procedures, including D2 lymphadenectomy, were performed according to the provisions of the 13th edition of the Japanese statutes on gastric cancer treatment (13). The main chemotherapy regimen for GNEC was XELOX (capecitabine plus oxaliplatin) or SOX (S1 plus oxaliplatin).

### Follow-Up

Postoperative follow-up assessments were performed every 3 months for 2 years and then every 6 months for 5 years.

### Statistical Analysis

All statistical analyses were performed using SPSS version 22.0 for Windows (SPSS Inc., Chicago, IL, USA). Categorical variables were analyzed using the chi-squared tests or Fisher exact tests, and continuous variables were analyzed using Student t-tests. The multivariable Cox proportional hazards regression models for OS included factors with a univariate p-value < 0.05, based on the log-rank test that compared Kaplan-Meier survival curves, as predictor variables. We performed multivariate analysis of variables that had a p-value <0.05 on univariate analysis. To simplify the nomogram, we adopted a stepwise forward regression approach (stepwise selection is a method of fitting the model, where the choice of predictive variables is made through an automated procedure) to screen out the variables most closely associated with the endpoint for improving its clinical utility. The prognostic abilities of the included factors and predictive models were compared by calculating the area under the curve (AUC). All tests were two-sided, and the statistical significance was set at a p value <0.05.

## Results

### Clinicopathological Characteristics

A total of 405 patients with GNEC were included in the training cohort including 7 centers from the Fujian province. Among the patients in the training group (n = 405), 301 (74.3%) were men, with an average age of 63.8 years; there were 40 cases, 105 cases, and 260 cases of pathological stage I, II, and III disease, respectively. A total of 117 patients (24.0%) had preoperative CEA level >5 ng/ml, and 251 patients (62.0%) had a Ki-67 index ≥60% (**Table 1** and **Table S1**).

**Table 1:** Clinicopathological characteristics.

	
Variable	n = 405
Sex	
pT stage	
T1	31
T2	41
T3	120
T4	213
N stage	
N0	93
N1	85
N2	101
N3a	89
N3b	37
pTNM	
I	40
II	105
III	260
Lymphovascular invasion	
No	218
Yes	168
Unknown	19
Nerve invasion	
No	263
Yes	127
Unknown	15
Pathological type	
NEC	218
MANEC	187
KI-67 index	
<60	127
≥60	251
Unknown	27

### Survival *A*nalysis

**Figure 1:**
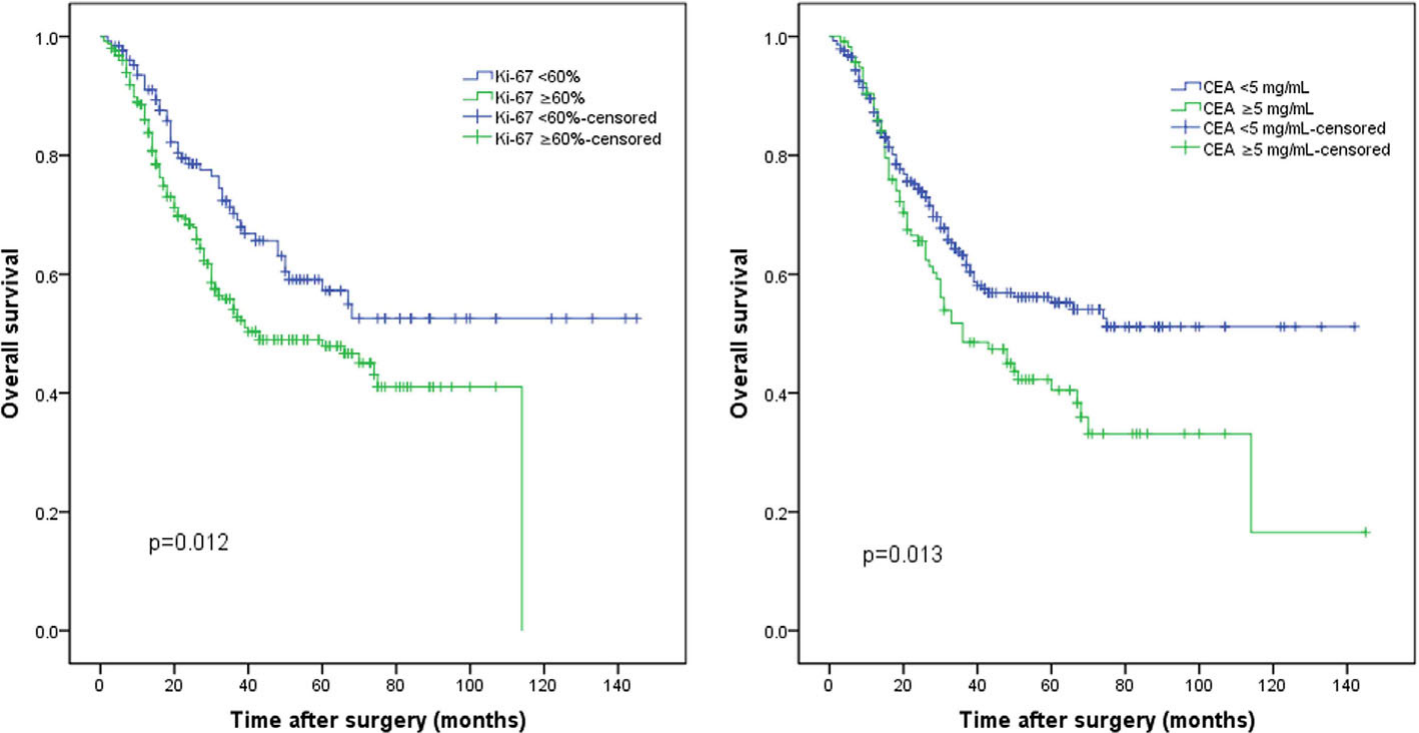
The whole cohort was grouped according to the **(A)** Ki-67 index and **(B)** preoperative carcinoembryonic antigen (CEA) levels.

**Figure 2:**
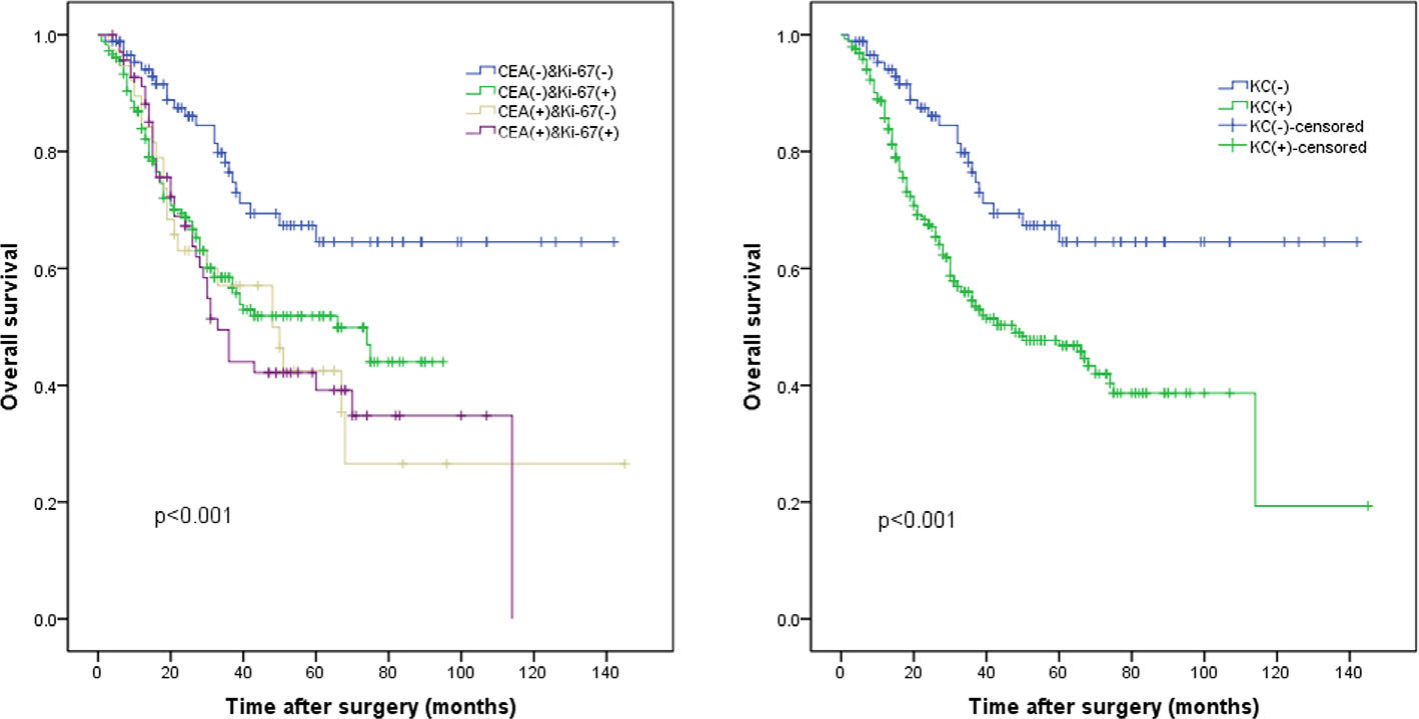
The whole cohort was divided into four subgroups according to **(A)** the KC status. **(B)** The KC(-) group (low Ki-67 index and normal carcinoembryonic antigen [CEA] levels) and the KC(+) group (high Ki-67 index or/and high CEA levels), p < 0.001.

The median follow-up period was 26.0 months (range, 1.0–145.0 months). The median Ki-67 index was used to classify the patients into a high Ki-67 index group (≥60%) and a low Ki-67 index group (<60%). The survival curve indicated that the 5-year OS rate was significantly worse in the high Ki-67 index group than in the low Ki-67 index group (47.9% vs. 57.2%, p = 0.012; **Figure 1A**). In addition, the 5-year OS rate was also significantly worse in the high CEA group (CEA ≥5 ng/ml) than in the normal CEA group (CEA <5 ng/ml; 40.5% vs. 55.2%, p = 0.013; **Figure 1B**). Patients were classified into four groups by combining the Ki-67 index and CEA level. Patients with both a low Ki-67 index and preoperative CEA levels had the best 5-year OS rates (64.6%), significantly better than those of the other three groups (p < 0.05); however, the differences between the survival rates of the other three groups were not statistically significant (**Figure 2A**).

We constructed a new prognostic indicator (KC) based on the Ki-67 index and CEA levels, and patients were divided into two groups: the KC(-) group (low Ki-67 index and normal CEA) and KC(+) group (elevated Ki-67 index and/or elevated CEA). The baseline information of the KC- and KC+ groups is shown in **Table S2**. The patient characteristics were significantly different, including the age, pT stage, pTNM stage, lymphovascular infiltration, and nerve infiltration, with all p-values < 0.05.

Survival curve analysis indicated that the long-term prognosis of the KC(+) group was significantly worse compared to that of the KC(-) group (64.6% vs. 46.8%, p < 0.001; **Figure 2B**).

### Univariate and Multivariate Analyses of Long-Term Prognosis

Univariate analysis revealed that a tumor size ≥5 cm, preoperative CEA level ≥5 ng/ml, Ki-67 index ≥60%, KC(+), and AJCC 8th stage were independent risk factors for poor long-term OS rates. Multivariate analysis revealed that only KC(+) and AJCC 8th stage were independent risk factors for poor OS rates in the whole group, with p < 0.05 for all (**Table 2**).

**Table 2:** Univariate and multivariate analyses of the factors associated with overall survival.

	Univariate analysis			Multivariate analysis		
	HR	95% CI	p-value	HR	95% CI	p-value
Sex						
Male	1.000					
Female	0.876	0.620–1.238	0.876			
Age (years)						
<65	1.000					
≥65	1.046	0.774–1.413	0.771			
Tumor diameter (cm)						
<5	1.000			–		
≥5	2.328	1.582–3.427	<0.001	–	–	–
CEA (ng/ml)						
<5	1.000			–		
>5	1.477	1.083–2.015	0.014	–	–	–
KI-67 index						
<60	1.000			–		
≥60	1.553	1.098–2.197	0.013	–	–	–
KC						
Negative	1.000			1.000		
Positive	2.208	1.417–3.441	<0.001	2.039	1.284–3.237	0.003
pTNM						
I	1.000			1.000		
II	3.376	1.016–11.225	0.047	4.448	1.049–18.859	0.043
III	10.383	3.303–32.637	<0.001	12.541	3.093–50.845	<0.001
Lymphovascular invasion						
No	1.000					
Yes	1.111	0.873–1.415	0.392			
Nerve invasion						
No	1.000					
Yes	1.052	0.811–1.366	0.700			
Adjuvant chemotherapy						
No	1.000					
Yes	1.204	0.870–1.666	0.262			

### Nomogram to Predict Long-Term Prognosis

**Figure 3:**
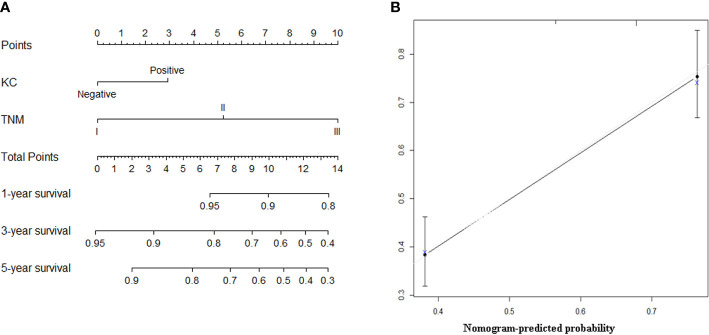
**(A)** The nomogram predicting overall survival of patients with gastric neuroendocrine carcinoma (GNEC). **(B)** The calibration curve of the nomogram for the training group.

Based on the results of multivariate analysis, KC status and AJCC 8th stage were combined to establish a nomogram that predicts the long-term prognosis of patients with GNEC. For predicting the long-term prognosis, the C-index for KC status (0.660, 95% CI: 0.621–0.700) was significantly higher than that of the AJCC 8th stage (0.635, 95% CI: 0.599–0.671; p = 0.005); the AUC value for KC status (0.700, 95% CI: 0.647–0.753) was also significantly higher than that of the AJCC 8th stage (0.675, 95% CI: 0.620–0.729). The calibration curves showed that the prediction performance of the nomogram was good (**Figures 3A, B**).

### External Validation

The clinicopathological characteristics of the validation group are shown in **Table S3**. As shown in **Figure S3**, the prognosis of the KC(+) group was significantly worse than that of the KC(-) group in the validation cohort (p = 0.002). In addition, the nomogram also showed a good predictive value, with a higher C-index (0.613, 95% CI: 0.569–0.659) than the AJCC 8th stage (0.578, 95% CI: 0.536–0.621, p = 0.028). The calibration curves for the validation cohort also showed good calibration (**Figure S4**).

## Discussion

To the best of our knowledge, this study is currently the largest study on GNEC and is the first in which a nomogram was prepared for GNEC prognosis. Thus, our study provided an important tool that can be used to evaluate the risk of death of patients with GNEC.

Compared to its role in other tumors, Ki-67 is a nuclear antigen that plays an important role in neuroendocrine tumors because of the increasing usage of histological grading systems and prognosis prediction for neuroendocrine tumors (10, 14). A high Ki-67 index was associated with poor progression-free survival and OS in neuroendocrine tumors (15, 16). Although patients with a Ki-67 index less than 55% showed a poor response to platinum-based chemotherapy, they had a better OS compared to those with a Ki-67 index of more than 55% (17). In the current study, the OS of the high Ki-67 index group was significantly worse than that of the low Ki-67 index group, according to a 60% Ki-67 threshold based on a ROC analysis. This finding supports the importance of the Ki-67 index for predicting the OS of patients with GNEC.

Little is known about the impact of CEA levels on the survival of patients with GNEC, even though CEA is a well-known prognostic factor for survival in patients with gastric adenocarcinoma (18). Recently, serum CEA levels have been recommended as a marker to evaluate the survival of patients with locoregional gastrin-independent gastric neuroendocrine tumors (19). However, the proportion of patients with high CEA levels in that study was 9.1% (6/66), significantly lower than the 28.9% (117/405) reported in the current study. This result indicates that the CEA level increases with the increase in tumor malignancy. In addition, the current study showed that the 5-year OS of the high CEA group was significantly worse than that of the normal CEA group. Accordingly, CEA levels could be used to stratify patients with GNEC into two distinct subgroups with high or low risks of death. Thus, the CEA level may help in providing individualized survival predictions for patients with GNEC and may be utilized to monitor treatment, tumor recurrence, and metastasis in patients with GNEC.

Most current studies on new prognostic indicators have shown significant baseline heterogeneity between groups, including differences in the CRP/prealbumin level, mean red blood cell volume, and modified Glasgow predictive scores (20–22). Even in the current study, patients in the KC(-) and KC(+) groups showed significant heterogeneity in tumor-related factors. However, on multivariate analysis that eliminated the effects of potential confounding factors, KC(+) was still closely associated with the long-term outcomes, confirming its prognostic value.

The ability of a staging system to predict the survival of a patient with GNEC after gastric resection has not yet been well validated. The European Neuroendocrine Tumor Society (ENETS) system is commonly used in European countries (23), and the AJCC staging system is widely accepted in North America  (24). In addition, the AJCC TNM system has a high prognostic value for grade 3 or mixed tumors (25). However, the prognostic accuracy is unclear and requires testing with a large cohort, which was done in the current study. Multivariate analysis revealed that KC(+) and AJCC 8^th^ stage were independent factors for OS. Thus, we constructed a nomogram based on KC(+) and AJCC 8^th^ stage to aid with clinical prognostic predictions and facilitate individualized evaluations of patients with GNEC. The calculated AUC (0.700) and C-index (0.660) showed that the performance of the nomogram was more powerful than that of the AJCC 8^th^ stage (C-index: 0.635, p = 0.005; AUC: 0.675, p = 0.020). Similar findings were observed in the validation cohorts.

The present study had some limitations. The possibility of selection bias cannot be excluded owing to the retrospective nature of the study. Moreover, we could not analyze recurrence-free survival because of inadequate data in some centers. In addition, adjuvant chemotherapy was not selected as a candidate factor because there was no uniform adjuvant therapy regimen; therefore, more efforts should be paid to normative treatment and data collection for these patients. Postoperative CEA decline is associated with better prognosis in patients with gastric cancer, but no studies in patients with GNEC have yet been published. Owing to the incomplete data regarding CEA levels in the current study, it was impossible to explore whether postoperative CEA level changes were closely associated with the prognosis of GNEC. The data about smoking habits were incomplete in this study. Therefore, we could not investigate the effects of smoking habits on the findings. Further larger cohorts with detailed information are warranted to validate the results.

In conclusion, we prepared a valid and reproducible nomogram that combined the TNM stage as well as the Ki-67 index and CEA levels; the nomogram may be an indispensable tool to help predict individualized risks of death and help clinicians manage patients with GNEC.

## Data Availability Statement

The datasets generated for this study are available on request to the corresponding author.

## Ethics Statement

The study was conducted in accordance with the Declaration of Helsinki and the Ethical Guidelines for Clinical Studies, and was approved by the institutional review boards of FMUUH (2018YF031-02). The patients/participants provided their written informed consent to participate in this study.

## Author Contributions

Conception and design: JX, YJZ, YZ, QH, HH, and C-MH. Provision of study materials or patients: All authors. Collection and assembly of data: All authors. Data analysis and interpretation: All authors. Manuscript writing: All authors. Accountable for all aspects of the work: All authors. All authors contributed to the article and approved the submitted version.

## Funding

This study was funded by the Scientific and Technological Innovation Joint Capital Projects of Fujian Province (2016Y9031), National Nature Science Foundation of China (No. 81871899), and Construction Project of Fujian Province Minimally Invasive Medical Center (No. [2017] 171). The second batch of special support funds was provided by the Fujian Province innovation and entrepreneurship talents (2016B013), QIHANG funds of Fujian Medical University (No. 2016QH025), Fujian province medical innovation project (2015-CXB-16), Fujian provincial health and family planning commission joint project (WKJ2016-2-27), and Chinese physicians association young physician respiratory research fund.

## Conflict of Interest

The authors declare that the research was conducted in the absence of any commercial or financial relationships that could be construed as a potential conflict of interest.

## Supplementary Material

Supplementary Figure 1 Flowchart showing the patient selection process.

Supplementary Figure 2 The **(A)** immunohistochemical, **(B)** synaptophysin and **(C)** chromogranin A for the diagnosis of gastric neuroendocrine carcinoma.

Supplementary Figure 3 The KC(-) group (low Ki-67 index and low carcinoembryonic antigen [CEA] levels) and the KC(+) group (high Ki-67 index and CEA levels) in the validation cohort, p = 0.002.

Supplementary Figure 4 The calibration curve of the nomogram for the validation group.
