# Supplementary figures and images for: Predictive Value of Combined Preoperative Carcinoembryonic Antigen Level and Ki-67 Index in Patients With Gastric Neuroendocrine Carcinoma After Radical Surgery
Source: Front Oncol. 2021 Mar 2;11:533039. doi: 10.3389/fonc.2021.533039 (PMC7962601; doi:10.3389/fonc.2021.533039)

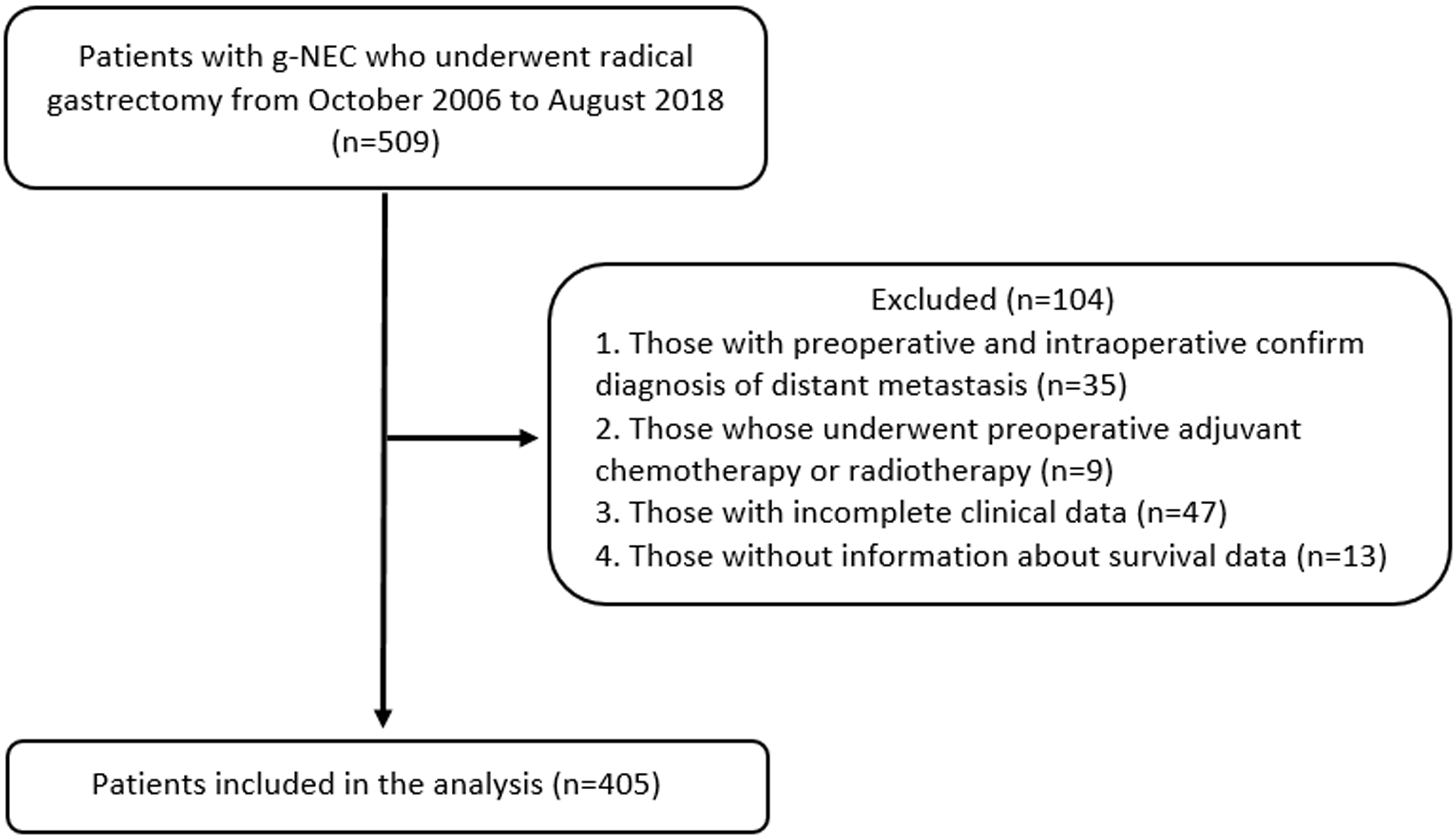

Supplement: Supplementary Figure 1 — Flowchart showing the patient selection process. [file Image_1.tif]

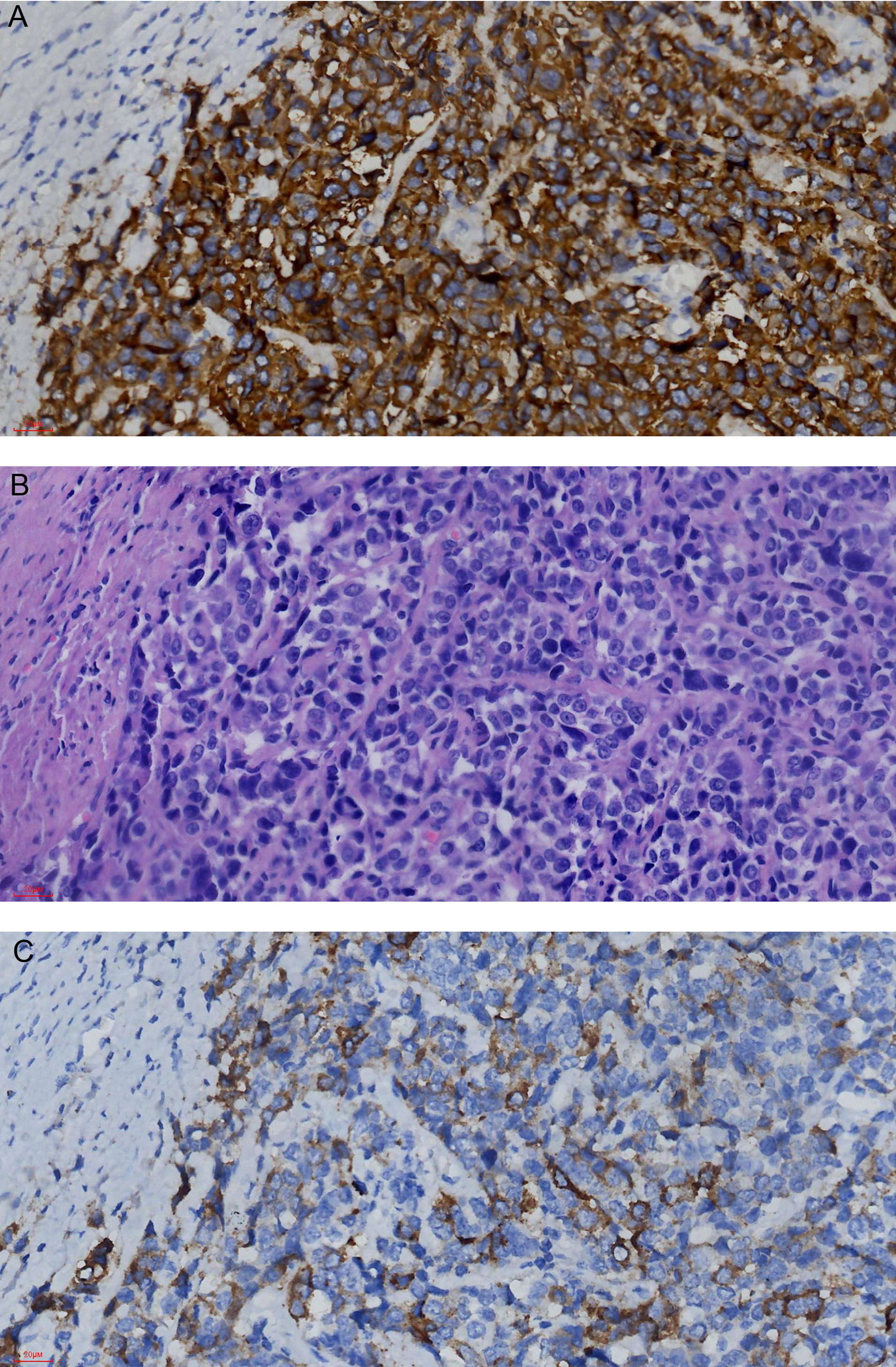

Supplement: Supplementary Figure 2 — The (A) immunohistochemical, (B) synaptophysin and (C) chromogranin A for the diagnosis of gastric neuroendocrine carcinoma. [file Image_2.tif]

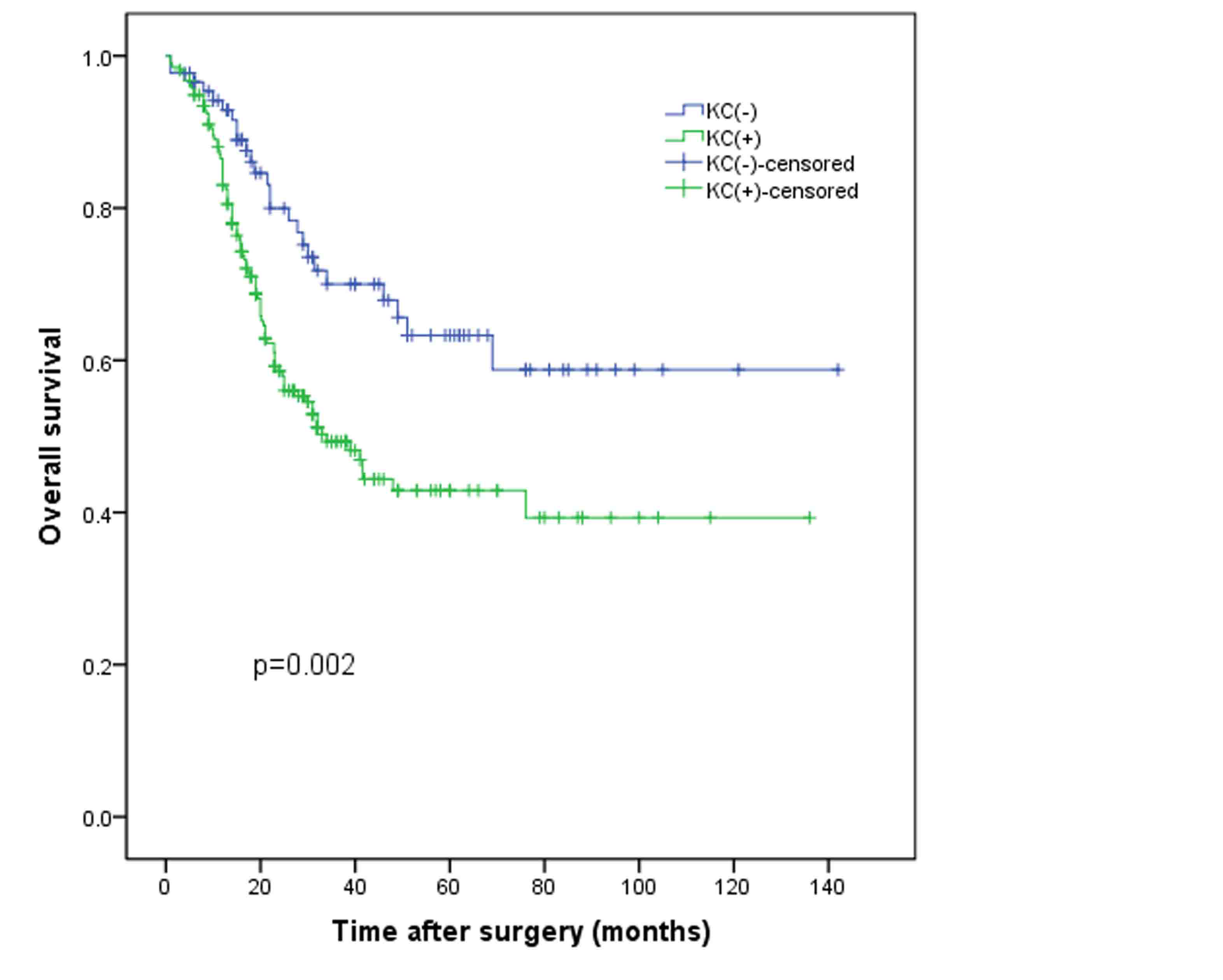

Supplement: Supplementary Figure 3 — The KC(-) group (low Ki-67 index and low carcinoembryonic antigen [CEA] levels) and the KC(+) group (high Ki-67 index and CEA levels) in the validation cohort, p = 0.002. [file Image_3.tif]

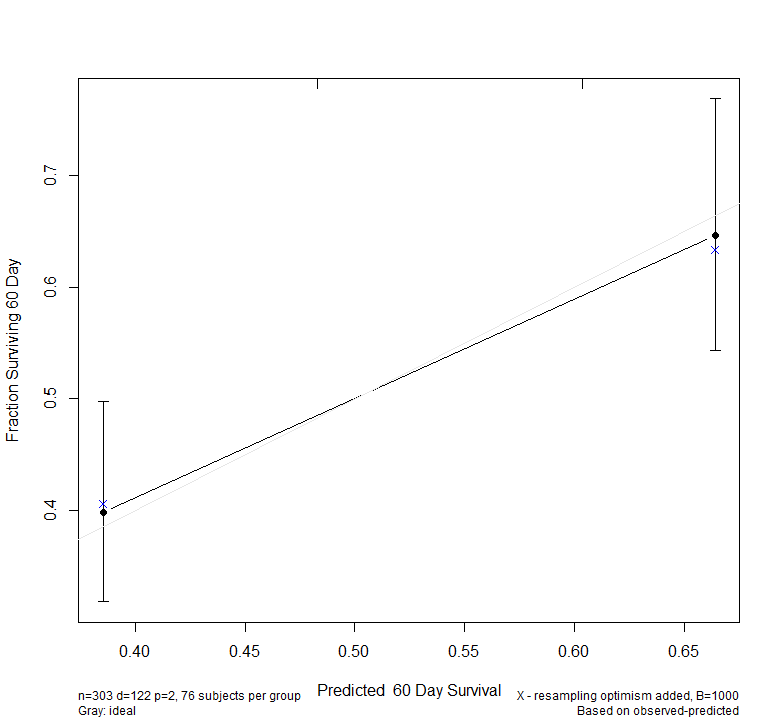

Supplement: Supplementary Figure 4 — The calibration curve of the nomogram for the validation group. [file Image_4.tiff]
