# Supplementary material for: Predictive Value of Combined Preoperative Carcinoembryonic Antigen Level and Ki-67 Index in Patients With Gastric Neuroendocrine Carcinoma After Radical Surgery
Source: Front Oncol. 2021 Mar 2;11:533039. doi: 10.3389/fonc.2021.533039 (PMC7962601; doi:10.3389/fonc.2021.533039)
Supplement: Supplementary file 5 [file Table_1.docx]

| **Table S1 Clinicopathological characteristics** | |
| --- | --- |
|  |  |
| Variable | n=405 |
| Sex |  |
| Male | 301 |
| Female | 104 |
| Age (years) | 63.8±9.3 |
| BMI (kg/m^2^) | 22.4±2.9 |
| Tumor diameter (cm) | 5.0±2.5 |
| ASA |  |
| <3 | 372 |
| ≥3 | 25 |
| Unknown | 8 |
| Tumor location |  |
| Upper | 202 |
| Middle | 78 |
| Low | 86 |
| Mix | 37 |
| Remnant stomach | 1 |
| Unknown | 1 |
| Hemoglobin (g/L) | 120.9±30.0 |
| Albumin (g/L) | 39.4±5.8 |
| Glucose (mmol/L) | 5.6±1.6 |
| CEA (ng/ml) |  |
| <5 | 308 |
| ≥5 | 117 |
| Lymphovascular invasion |  |
| No | 218 |
| Yes | 168 |
| Unknown | 19 |
| Nerve invasion |  |
| No | 263 |
| Yes | 127 |
| Unknown | 15 |
| Surgical method |  |
| Open | 175 |
| Laparoscopic | 229 |
| Others | 1 |
| Gastrectomy extent |  |
| Total | 309 |
| Distal | 80 |
| Proximal | 12 |
| Others | 4 |
| Reconstruction |  |
| B-1 | 30 |
| B-II | 73 |
| Roux-en-Y | 268 |
| Others | 30 |
| Surgical duration (min) | 204.0±60.6 |
| Complications |  |
| No | 213 |
| Yes | 191 |
| Unknown | 1 |
| Adjuvant chemotherapy |  |
| No | 143 |
| Yes | 262 |
