# Supplementary material for: Predictive Value of Combined Preoperative Carcinoembryonic Antigen Level and Ki-67 Index in Patients With Gastric Neuroendocrine Carcinoma After Radical Surgery
Source: Front Oncol. 2021 Mar 2;11:533039. doi: 10.3389/fonc.2021.533039 (PMC7962601; doi:10.3389/fonc.2021.533039)
Supplement: Supplementary file 6 [file Table_2.doc]

| **Table S2 Clinicopathological characteristics according to different KC status.** | | | |
| --- | --- | --- | --- |
|  |  |  |  |
|  | KC(-)  (n=89) | KC(+) (n=289) | p-value |
| Gender |  |  | 0.418 |
| Male | 63 | 217 |  |
| Female | 26 | 72 |  |
| Age (years) | 61.7±10.3 | 64.4±9.0 | 0.018 |
| Tumor diameter (cm) | 4.6±2.6 | 5.1±2.4 | 0.061 |
| Tumor location |  |  | 0.444 |
| Upper | 40 | 146 |  |
| Middle | 24 | 51 |  |
| Low | 18 | 64 |  |
| Mix | 7 | 26 |  |
| Remnant stomach | 0 | 1 |  |
| pT stage |  |  | 0.026 |
| T1 | 13 | 16 |  |
| T2 | 9 | 30 |  |
| T3 | 21 | 95 |  |
| T4 | 46 | 148 |  |
| N stage |  |  | 0.050 |
| N0 | 29 | 59 |  |
| N1 | 22 | 55 |  |
| N2 | 18 | 75 |  |
| N3a | 14 | 69 |  |
| N3b | 6 | 31 |  |
| pTNM |  |  | 0.016 |
| I | 14 | 23 |  |
| II | 29 | 72 |  |
| III | 46 | 194 |  |
| Lymphovascular invasion |  |  | 0.017 |
| No | 60 | 149 |  |
| Yes | 25 | 130 |  |
| Unknown | 4 | 10 |  |
| Nerve invasion |  |  | 0.036 |
| No | 67 | 181 |  |
| Yes | 22 | 98 |  |
| Unknown | 0 | 10 |  |
| Adjuvant chemotherapy |  |  | 0.319 |
| No | 35 | 97 |  |
| Yes | 54 | 192 |  |
