# Supplementary material for: Predictive Value of Combined Preoperative Carcinoembryonic Antigen Level and Ki-67 Index in Patients With Gastric Neuroendocrine Carcinoma After Radical Surgery
Source: Front Oncol. 2021 Mar 2;11:533039. doi: 10.3389/fonc.2021.533039 (PMC7962601; doi:10.3389/fonc.2021.533039)
Supplement: Supplementary file 7 [file Table_3.doc]

| **Table S3 Clinicopathological characteristics of the validation group.** | |
| --- | --- |
|  |  |
| Variable | n=305 |
| Gender |  |
| Male | 238 |
| Female | 67 |
| Age (years) | 63.9±9.3 |
| BMI (kg/m^2^) | 23.2±3.2 |
| Tumor diameter (cm) | 4.9±3.2 |
| ASA |  |
| <3 | 216 |
| ≥3 | 34 |
| Unknown | 55 |
| Tumor location |  |
| Upper | 139 |
| Middle | 60 |
| Low | 88 |
| Mix | 14 |
| Remnant stomach | 1 |
| Unknown | 3 |
| CEA (ng/ml) |  |
| <5 | 219 |
| ≥5 | 86 |
| pT stage |  |
| T1 | 20 |
| T2 | 29 |
| T3 | 12 |
| T4 | 244 |
| N stage |  |
| N0 | 97 |
| N1 | 63 |
| N2 | 75 |
| N3a | 58 |
| N3b | 12 |
| pTNM |  |
| I | 30 |
| II | 86 |
| III | 189 |
| Lymphovascular invasion |  |
| No | 186 |
| Yes | 107 |
| Unknown | 12 |
| Nerve invasion |  |
| No | 214 |
| Yes | 76 |
| Unknown | 15 |
| Surgical method |  |
| Open | 242 |
| Laparoscopic | 57 |
| Others | 6 |
| Gastrectomy extent |  |
| Total | 149 |
| Distal | 92 |
| Proximal | 34 |
| Others | 30 |
| Reconstruction |  |
| B-1 | 58 |
| B-II | 33 |
| Rou-en-Y | 193 |
| Others | 21 |
| Surgical durations(min) | 191.8±217.5 |
| Pathological type |  |
| NEC | 160 |
| MANEC | 145 |
| KI-67% |  |
| <60 | 113 |
| ≥60 | 192 |
| Complications |  |
| No | 206 |
| Yes | 22 |
| Unknown | 77 |
| Adjuvant chemotherapy |  |
| No | 141 |
| Yes | 164 |
